# Le diagnostic anténatal de la trisomie 21 par l'hybridation in situ en fluorescence (FISH): à propos des premiers tests réalisés au Maroc

**Published:** 2012-10-22

**Authors:** Afaf Lamzouri, Abdelhafid Natiq, Mariam Tajir, Mohamed Sendid, Abdelaziz Sefiani

**Affiliations:** 1 Centre de génomique humaine, Faculté de Médecine et Pharmacie, Université Mohammed V Souissi, Rabat, Maroc; 2 Département de génétique médicale, Institut National d'Hygiène, Rabat, Maroc; 3 Faculté des sciences, Université Mohammed V Agdal, Rabat, Maroc; 4 Clinique la Capitale, Rabat, Maroc

**Keywords:** Trisomie 21, diagnostic anténatal, Hybridation In Situ en Fluorescence, Maroc, Afrique, Trisomy 21, prenatal diagnosis, Fluorescence in Situ Hybridization, Morocco, Africa

## Abstract

**Introduction:**

Le but de cette étude était de présenter les premiers résultats de diagnostic anténatal de la trisomie 21 par la technique d'hybridation in situ en fluorescence (FISH) au Maroc et discuter son intérêt dans le diagnostic rapide de cette aneuploïdie.

**Méthodes:**

Ce travail a été réalisé chez 23 femmes avec des grossesses à haut risque de trisomie 21. La moyenne d’âge des gestantes étaient de 37,43 ans avec des extrêmes de 21 et 43 ans. Toutes étaient musulmanes mariées, mariage légitimé par la Charia, dont trois mariages consanguins, sauf une originaire de la République Démocratique du Congo qui était chrétienne et concubine. La majorité des femmes étaient fonctionnaires et avaient un niveau de scolarisation moyen à élevé. Toutes les patientes ont bénéficié d'une consultation de génétique médicale au cours de laquelle il leur a été donné des informations sur la technique, son intérêt et ses limites. Il s'agit de femmes enceintes qui avaient soit un âge maternel élevé ou des signes d'appel échographiques et/ ou biochimiques. Une des patientes était porteuse d'une translocation robertsonienne t(14;21) équilibrée. Une amniocentèse a été réalisée chez toutes les gestantes et aucun avortement n'a était induit par ce geste invasif. L’âge gestationnel moyen à la première consultation était de 14 semaines d'aménorrhée (SA) et à l'amniocentèse était de 16 SA et 5 jours. L'analyse FISH a été réalisée, après consentement des couples, sur des cellules non cultivées à partir des échantillons de liquides amniotiques, en utilisant des sondes spécifiques du chromosome 21.

**Résultats:**

Parmi les 23 patientes qui ont bénéficiées d'un diagnostic anténatal de la trisomie 21 par la technique FISH, nous avons pu rassurer 21 d'entre elles, et nous avons détecté deux cas de trisomie 21 fœtal.

**Conclusion:**

La technique FISH permet un diagnostic anténatal rapide, en moins de 48h, de la trisomie 21 sur une faible quantité de liquide amniotique. Elle offre aux couples l'avantage de prendre, dans des délais raisonnables, la décision qui leur convient concernant la poursuite ou non de la grossesse. Elle permet souvent, avec un résultat normal, de rassurer rapidement les femmes enceintes trop angoissées.

## Introduction

Le Maroc est un pays arabo-berbère musulman, situé à l′angle Nord-Ouest du continent Africain. Il compte 32,3 millions d′habitants [1] et sa superficie est d′environ 710.850 Km^2^. Le taux de scolarisation a augmenté considérablement au Maroc, en 2011, 97,5% des enfants en âge de scolarité ont accès aux écoles primaires [2], et le taux de chômage a reculé de 8,1% en 2012 [3]. Le système national de santé au Maroc est organisé en 3 secteurs, un secteur privé à but lucratif, un secteur privé à but non lucratif et un secteur public comprenant un réseau hospitalier public, un réseau de soins de santé de base, des instituts et laboratoires nationaux, des services de santé des forces armées royales, et des bureaux municipaux et communaux d'hygiène. Le premier centre de génétique médicale a été crée au Maroc à l'Institut National d'Hygiène en 1989, d'autres services et laboratoires de génétique ont était progressivement mis en place à l'Institut Pasteur et dans les quatre centres hospitaliers universitaires du Maroc. Les maladies rares dont 80% sont d'origine génétiques ne font pas partie des priorités du système de santé marocain, ainsi peu d’études épidémiologiques sont réalisés dans ce domaine, mais du fait du taux élevé de la consanguinité au Maroc qui est de 15.25% [4] les maladies génétiques, en particulier celles transmises selon le mode autosomique récessif, constituent un véritable problème de santé publique.

La trisomie 21 est l'anomalie chromosomique viable la plus fréquente chez l'homme, sa prévalence est estimée entre 1,4 et 2 pour 1000 grossesses [5]. Elle représente la principale cause génétique de retard mental et elle est à l'origine d'un handicap social important [6].

Au Maroc Le manque de structures d′accueil condamne les enfants trisomiques 21, à l'instar de nombreux autres enfants porteurs d'un handicap d'origine génétique, à l′isolement et à la marginalisation. Certaines associations montent au créneau pour tenter de combler ce vide en œuvrant à l′intégration de ces enfants dans la société. C′est notamment le cas de l'Association Marocaine de Soutien et d′Aide aux Handicapés Mentaux (AMSHAM) à Rabat et l'association marocaine pour enfants trisomique 21 (AMET 21) à Meknès. Mais leurs ressources humaines et matérielles demeurent limitées face à une demande élevée.

La recherche d'une trisomie 21 fœtal est le diagnostic prénatal (DPN) le plus fréquemment réalisé au monde. Il est indiqué devant des signes d'appel échographiques fœtaux, un dépistage sérique anormal chez la mère, des anomalies chromosomiques lors d'une grossesse antérieure, devant une translocation équilibrée impliquant le chromosome 21 chez un des parents, ou lorsque l’âge maternel dépasse les 38 ans.

En cas de risque élevé de trisomie 21, une amniocentèse est proposée aux parents, afin de réaliser un caryotype fœtal sur liquide amniotique [7]. Le principal inconvénient de cette technique, est la nécessité d'une culture cellulaire avant l'analyse qui prend 2 à 3 semaines sur une ponction d'au moins 20 ml de liquide amniotique, un taux d′échec de la culture d′environ 1% et un risque de contamination cellulaire non négligeable [8, 9]. Ce temps requis pour réaliser l′analyse chromosomique constitue un lourd fardeau clinique et psychologique pour le couple. L'hybridation in situ en fluorescence (FISH), qui est une technique de cytogénétique moléculaire, a l'avantage de détecter rapidement cette aneuploïdie par des sondes chromosomiques spécifiques, sur des cellules non cultivées, en moins de 48h et sur une faible quantité de liquide amniotique, ainsi elle offre aux couples l'avantage de prendre, dans des délais raisonnables, la décision qui leur convient concernant la poursuite ou non de la grossesse. Par ailleurs au Maroc, il n'y a pas à ce jour de cadre juridique qui encadre les maladies génétique et la pratique du diagnostic prénatal, et toute interruption de grossesse est bannie par la loi, sauf en cas de risque pour la vie de la mère.

L′objectif de ce travail est de présenter nos premiers résultats de diagnostic anténatal de la trisomie 21 par la technique FISH au Maroc et de discuter son intérêt dans le diagnostic rapide de cette aneuploïdie.

## Méthodes

Il s'agit d'un travail prospectif réalisé entre juin 2010 et mai 2011, au département de Génétique Médicale à l'Institut National d'Hygiène de Rabat et qui rapporte les premières observations de diagnostic anténatal de la trisomie 21 par la technique FISH au Maroc.

**Figure 1:**
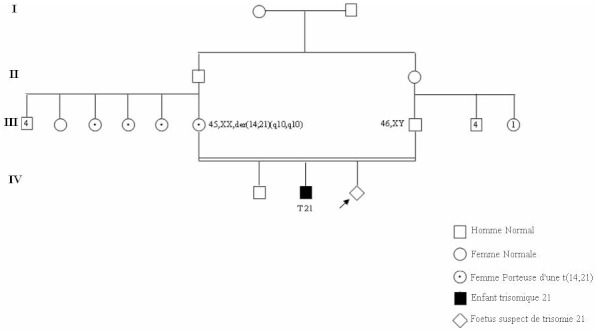
Arbre généalogique de la patiente 13 illustrant une translocation familiale t(14;21)

### Critères d'inclusion des femmes enceintes

**Figure 2:**
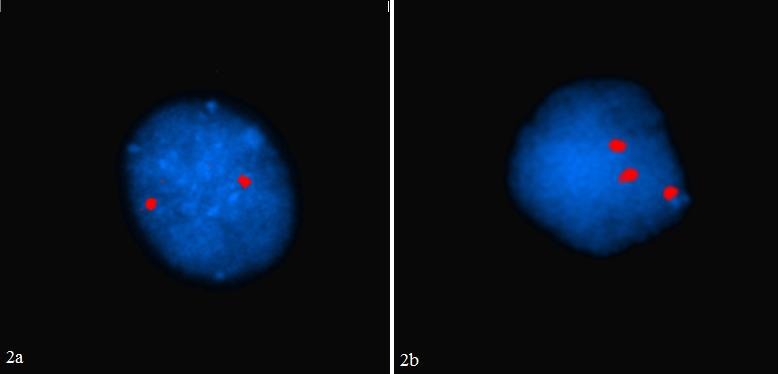
(a) Amniocyte avec de deux spots rouges spécifiques des chromosomes 21; (b) Amniocyte avec trois spots rouges spécifiques des chromosomes 21

Ce travail a été réalisé chez 23 femmes adressées à la consultation de génétique par leurs médecins gynécologues pour une grossesse à risque de trisomie 21. Il s'agit de patientes qui avaient soit un âge maternel élevé ou des signes d'appel échographiques et/ ou biochimiques avec des valeurs anormales des marqueurs sériques dans le sang maternel. Une des patientes (cas n°13) avait un risque de trisomie 21 très élevé, car elle était porteuse d'une translocation robertsonienne t(14;21) équilibrée (voir arbre généalogique Figure 2).

### Consultation pré DPN et consentement

Toutes les patientes ont bénéficié d'une consultation de génétique pré DPN et ont été vu par un médecin généticien de notre équipe. Au cours de cette consultation, des informations et des explications ont été données aux parents sur les risques liés à l'amniocentèse, la nature des anomalies recherchées, les limites techniques de la FISH et sur les possibilités du suivi offertes au Maroc. Le consentement des couples étaient requis.

### Réception et enregistrement du prélèvement

L'analyse a été effectuée après un rendez-vous coordonné entre le médecin généticien et le gynécologue. Un volume moyen de 4 ml de liquide amniotique a été ponctionné puis acheminer au laboratoire dans un tube a fond conique de 15 ml, accompagné d'une fiche de liaison remplie par le médecin traitant. A son arrivée au laboratoire, les échantillons ont été enregistrés et un dossier médical a été ouvert pour chaque patiente comportant tous les renseignements cliniques, biologiques et échographiques.

### Technique FISH et analyse des résultats

Le protocole qui a été utilisé lors de cette étude est certifié « guide des bonnes exécutions d'analyses» GBEA avec légère modification pour l'adapter aux conditions de notre département. L'analyse FISH a été réalisée sur des cellules interphasiques à partir d’échantillons de liquides amniotiques: après la préparation de cellules amniotiques non cultivées et l’étalement sur glace, un prétraitement des lames, suivi d'une dénaturation-hybridation de l′ADN des échantillons et de la sonde ont été réalisés. La sonde commerciale utilisée est une sonde locus spécifique d'environ 200 kb, localisée en 21q22.13-q22.2 (région critique du syndrome de down DSCR) contenant les marqueurs D21S259, D21S341 et D21S342 (ABBOTT). Après un lavage post-hybridation, la lecture a été faite avec un microscope à fluorescence NIKON E600, et l'analyse des résultats a été réalisée avec un logiciel de capture et d'analyse d'image de type Cytovision version 3.93.

Environ cents noyaux par lame ont été analysés: un minimum de 95% de noyaux analysables comportant 2 signaux est requis pour qu'un résultat soit considéré normal. 70% des noyaux analysables doivent comporter 3 signaux pour accepter le diagnostic de trisomie. Pour la validation des résultats, nous avons réalisé une double lecture aveugle des lames.

## Résultats

Dans notre série, la moyenne d’âge des gestantes étaient de 37,43 ans avec des extrêmes de 21 et 43 ans. Toutes étaient musulmanes mariées, mariage légitimé par la Charia, dont trois mariages consanguins, sauf une originaire de la République Démocratique du Congo qui était chrétienne et concubine. La majorité des femmes étaient fonctionnaires et avaient un niveau de scolarisation moyen à élevé. Toutes les patientes étudiées avaient des grossesses à haut risque de trisomie 21 et avaient toutes au moins une indication du diagnostic anténatal. L’âge maternel était = 38 ans dans 73,9% des cas. 21,7% des patientes avaient une clarté nucale supérieure à 3 mm, 86,9% des cas avaient un risque de trisomie 21 fœtal estimé par triple test compris entre 1/10 et 1/150 et une gestante avait un risque élevé lié à une translocation robertsonienne familiale t(14;21).

Parmi les 23 patientes chez qui un DPN de trisomie 21 rapide par FISH a été réalisé, nous avons pu rassurer 21 femmes, car nous avons détecté uniquement deux chromosomes 21 (amniocytes avec deux spots rouges spécifiques des chromosomes 21; Figure 2 (a)). Chez deux patientes la FISH a mis en évidence une trisomie 21 fœtale (trois spots rouges, sur les 100 noyaux analysés; Figure 2 (b)).

La trisomie 21 fœtale a été confirmée chez une première patiente qui avait trois indications du DPN: un âge élevé de 42 ans, une clarté nucale de 5,5 mm nettement supérieure au seuil pathologique et un risque estimé à 1/10 au triple test.

La deuxième patiente chez qui la technique FISH a confirmé l'atteinte fœtale était âgée de 38 ans et était suivie dans notre service car elle était porteuse d'une translocation robertsonienne familiale équilibrée t(14;21), découverte suite à la naissance d'un premier enfant trisomique 21, et qui lui donnait un risque de récurrence estimé à 20%. De par ce risque élevé et son impact psychologique, la situation de ce couple constitue une bonne indication au diagnostic préimplantatoire (DPI).

Le Tableau 1 résume les caractéristiques cliniques, paracliniques et les résultats des 23 cas étudiés. Dans notre série, seules trois patientes ont souhaité réaliser en parallèle un caryotype fœtal dont le résultat normal a confirmé celui obtenu par FISH. Les autres patientes, chez qui la FISH était normale, n'ont pas fait de caryotype fœtal et ont toutes donné naissance à des nouveau-nés non trisomiques 21. En ce qui concerne l'issue des deux grossesses avec fœtus trisomique 21 confirmé par FISH, une patiente a préféré ne pas communiquer sa décision, alors que la deuxième, mère déjà d'un premier enfant trisomique 21, a réalisé un avortement clandestin car l'interruption volontaire de grossesse au Maroc est illégale.

**Tableau 1:** Caractéristiques et résultats des patientes incluses dans l’étude

Cas	Age maternel	Age de grossesse (SA)	Clarté nucale (mm) à 12 SA	Risque estimé par triple test	Antécédents particuliers	Résultat
1	42	17	5,5	1/10	-	Trisomie 21
2	38	17	1	1/50	-	Normal
3	40	18	2,9	1/150	-	Normal
4	41	20	2,9	1/140	-	Normal
5	40	17	-	1/10	-	Normal
6	39	14	-	1/50	-	Normal
7	21	17	3,2	1/10	-	Normal
8	40	14	-	1/60	-	Normal
9	41	14	2,5	1/10	-	Normal
10	42	21	-	1/10	-	Normal
11	29	18	3,7	Non fait	-	Normal
12	41	16	2,8	1/10	-	Normal
13	38	14	2,8	Non fait	t (14;21) maternelle	Trisomie 21
14	41	14	3,1	1/60	-	Normal
15	43	18	2	1/40	-	Normal
16	32	16	2	1/10	-	Normal
17	41	17	2,5	1/10	-	Normal
18	41	18	2,4	1/20	-	Normal
19	31	17	0,8	1/110	-	Normal
20	26	16	3,2	Non fait	-	Normal
21	43	17	-	1/60	-	Normal
22	28	18	2,6	1/50	-	Normal
23	43	19	2,8	1/140	-	Normal

## Discussion

Les pratiques de dépistage d'un risque accru de trisomie 21 chez la femme enceinte varient d'un centre de dépistage à l'autre. Différentes recommandations ont été émises par les sociétés savantes. En 2004, le comité scientifique de l'International Down Syndrome Screening Group a conclu [10] que le dépistage de la trisomie 21 sur le seul critère d’âge n’était plus justifiable. En 2007, L'American College of Obstetricians and Gynecologists en association avec la Society for Maternal Fetal Medecine [11] recommandent pour le dépistage de la trisomie 21 la mesure de la clarté nucale et le dosage des marqueurs sériques du premier trimestre. Un ensemble de trois marqueurs, désigné sous le nom de triple test, comprend la gonadotrophine chorionique humaine (HCG), l′oestriol (UE3) et l′alpha-foetoprotéine (AFP) est le plus utilisé [12]. Le taux de ces marqueurs interprété en fonction de paramètres maternels (âge de la mère, poids, tabagisme…) permet d’établir un score évaluant le risque de trisomie 21 fœtale. Le diagnostic anténatal est indiqué lorsque ce risque est supérieur à 1/250.

Au Maroc, l'utilisation très répandu aujourd'hui en gynécologie du triple test, génère une grande angoisse chez les couples pouvant parfois conduire à tort à une interruption volontaire de grossesse, vu que ce test biologique est perçu comme un moyen de diagnostic de trisomie 21 et non une simple estimation d'un risque qui permet d'identifier les femmes enceintes nécessitant un diagnostic anténatal.

L'avantage majeur de la technique FISH proposée à nos patientes est la possibilité de rendre très rapidement, en 24 à 48h, des résultats, majoritairement rassurants, à des couples très angoissés et souvent mal informés sur la signification exacte du triple test. La majorité des femmes avaient compris à tort que ce test permettait le diagnostic et non l'estimation de risque.

Le diagnostic anténatal des aneuploïdies fœtales est faisable dans d'autres pays africains comme le Cameroun, le Nigeria et l'Afrique du Sud, et des études similaires à la notre ont été réalisé en Egypte et en Tunisie avec des échantillons plus larges en utilisant plusieurs techniques y compris la technique FISH [13, 14].

Habituellement, la recherche de la trisomie 21 en cas de risque élevé de survenue d'aneuploïdies, se fait par caryotype fœtal sur cellules amniotiques, technique longue, nécessitant, selon les laboratoires, entre deux et trois semaines de délai avant le rendu du résultat, ce qui allonge la durée du stress et d'anxiété chez les couples à risque [15]. Un moyen de diagnostic plus rapide est important, surtout lorsque l’échographie détecte des anomalies morphologiques et/ou que le stade de la grossesse est avancé. C'est ce que permet l'hybridation in situ en fluorescence sur cellules amniotiques en interphase, car elle présente l'avantage de ne pas nécessiter de culture cellulaire. L'utilisation d'une sonde spécifique du chromosome 21 sur des noyaux interphasiques d'amniocytes non cultivés en comptant le nombre de spots fluorescents, permet de confirmer ou d'infirmer le diagnostic d'une trisomie 21 en 24 à 48h. La sensibilité et la spécificité sont bonnes et la corrélation entre les résultats de la FISH et ceux du caryotype classique est supérieure à 99% pour la plupart des séries publiées [8, 16].

L'amplification en chaîne par polymérase fluorescente quantitative (QF-PCR) est une méthode plus récente qui peut être utilisée pour déterminer le nombre de copies d'une séquence d'ADN. Elle est aussi fiable et précise que la FISH et le caryotypage en ce qui concerne les aneuploïdies ciblées. Le principale inconvénient de cette technique est celui de la contamination par des cellules maternelles ce qui donnerait des faux négatifs [17].

Par ailleurs, Les limites de la FISH interphasique sont d'ordre technique et clinique: liés à la nature du prélèvement, 10 à 15% des échantillons sont ininterprétables ou non informatifs (contamination maternelle, prélèvement hémorragique, liquide amniotique tardif…). Dans notre série, 11 échantillons de liquides amniotiques sur les 23 étaient légèrement hémorragiques, mais après traitement adapté, on n'a pas eu de difficultés d'analyse liées à la contamination par des cellules maternelles. Cette technique a également l'inconvénient de ne pas permettre de distinguer une trisomie 21 libre (accidentelle) d'une trisomie 21 par translocation robertsonienne.

Pour toutes ces raisons, même si la concordance avec le caryotype est de presque 100%, certains auteurs préconisent de compléter l’étude FISH intérphasique par un caryotype fœtal de confirmation avant de pratiquer une interruption de grossesse.

Au Maroc, il n'y a pas à ce jour de cadre juridique qui encadre la pratique du diagnostic prénatal, et toute interruption de grossesse est bannie par la loi, sauf en cas de risque pour la vie de la mère. Devant une demande croissante pour le DPN, Il devient indispensable de mettre en place un Comité National de Bioéthique, qui pourra se pencher sur l'organisation des techniques et des structures habilitées à réaliser des DPN. Il pourra également réfléchir sur le statut de l'embryon et donner un avis sur l'interruption médicale de grossesse en cas de fœtus porteurs d'anomalies génétiques graves.

## Conclusion

Le diagnostic anténatal de la trisomie 21 par la technique FISH est possible au Maroc. Cette technique permet un diagnostic rapide, en moins de 48h, de la trisomie 21 sur une faible quantité de liquide amniotique d'environ 4 ml. Elle offre aux couples l'avantage de prendre, dans des délais raisonnables, la décision qui leur convient concernant la poursuite ou non de la grossesse. Elle permet souvent, avec un résultat normal, de rassurer rapidement les femmes enceintes trop angoissées.
